# Effect of liberal or conservative oxygen therapy on the prognosis for mechanically ventilated intensive care unit patients: a meta-analysis

**DOI:** 10.1590/1516-3180.2021.0062.21092021

**Published:** 2022-04-02

**Authors:** Wei-Hua Dong, Wen-Qing Yan, Zhi Chen

**Affiliations:** Undergraduate Student, Emergency Department, Jiangxi Provincial People’s Hospital Affiliated to Nanchang University; Medical Department of Nanchang University, Nanchang, Jiangxi, China.; II Undergraduate Student, Emergency Department, Jiangxi Provincial People’s Hospital Affiliated to Nanchang University; Medical Department of Nanchang University, Nanchang, Nanchang, Jiangxi, China.; III MD. Chief Physician, Emergency Department, Jiangxi Provincial People’s Hospital Affiliated to Nanchang University, Nanchang, Jiangxi, China.

**Keywords:** Respiration, artificial, Oxygen, Adverse effects [subheading], Conservative oxygen therapy, Critically ill, Meta-analysis

## Abstract

**BACKGROUND:**

For critically ill patients, physicians tend to administer sufficient or even excessive oxygen to maintain oxygen saturation at a high level. However, the credibility of the evidence for this practice is unclear.

**OBJECTIVE:**

To determine the effects of different oxygen therapy strategies on the outcomes of mechanically ventilated intensive care unit (ICU) patients.

**DESIGN AND SETTING:**

Systematic review of the literature and meta-analysis conducted at Jiangxi Provincial People’s Hospital, Affiliated to Nanchang University, Nanchang, China.

**METHODS:**

We systematically searched electronic databases such as PubMed and Embase for relevant articles and performed meta-analyses on the effects of different oxygen therapy strategies on the outcomes of mechanically ventilated ICU patients.

**RESULTS:**

A total of 1802 patients from five studies were included. There were equal numbers of patients in the conservative and liberal groups (n = 910 in each group). There was no significant difference between the conservative and liberal groups with regard to 28-day mortality (risk ratio, RR = 0.88; 95% confidence interval, CI = 0.59-1.32; P = 0.55; I^2^ = 63%). Ninety-day mortality, infection rates, ICU length of stay, mechanical ventilation-free days up to day 28 and vasopressor-free days up to day 28 were comparable between the two strategies.

**CONCLUSIONS:**

It is not necessary to use liberal oxygen therapy strategies to pursue a higher level of peripheral oxygen saturation for mechanically ventilated ICU patients. Conservative oxygen therapy was not associated with any statistically significant reduction in mortality.

## INTRODUCTION

Mechanical ventilation (MV) is a common support intervention in intensive care units (ICUs). More than half of ICU patients receive mechanical ventilation on admission.^1^ It has been estimated that 2-3 million ICU patients receive MV annually around the world.^2,3^ Respiratory failure is the main indication for MV among ICU patients.^4^ Oxygen therapy is an important treatment for these patients.

Myocardial hypoxia was first identified as being responsible for angina in 1928.^5^ Oxygen therapy, a harmless, potentially beneficial therapeutic modality, is becoming increasingly used in clinical practice. In traditionally liberal oxygen therapy, most patients are given oxygen exceeding the physiological level because of fear of tissue hypoxia.^6,7^ Some patients, even without hypoxemia, are given oxygen therapy prophylactically for prevention of tissue hypoxia. A large population of mechanically ventilated ICU patients is exposed to hyperoxia.^8^ When arterial oxygen partial pressure is on the flat part of the oxygen hemoglobin dissociation curve, high concentrations of oxygen do not increase oxygen delivery significantly, even if these can increase the partial pressure of oxygen markedly, according to the characteristics of oxygen hemoglobin dissociation.^9^

Hyperoxia can also cause potential harm to patients.^10^ It can lead to lung interstitial fibrosis, tracheobronchitis, alveolar protein leakage, neutrophil infiltration,^11-13^ impaired immune function, ^14^ increased vascular resistance, reduced cardiac output^15^ and large quantities of free radicals. ^16^ In view of this, it has been proposed that conservative oxygen therapy strategy should be used^17^ in order to avoid unnecessary hyperoxia while ensuring oxygen delivery. Several studies have indicated that conservative oxygen therapy improves the prognosis for ischemic stroke and myocardial infarction.^18,19^

Despite this, the guidelines available regarding oxygen therapy standards and targets are contradictory and inconsistent.^20-22^ Studies on this topic have evaluated the effects of different oxygen therapy strategies on the prognosis for mechanically ventilated patients. However, the conclusions that they reached have not been completely coherent.^17,23-26^ Therefore, we decided to conduct a secondary analysis.

## OBJECTIVE

We performed a systematic review of the literature to determine the effects of different oxygen therapy strategies on the outcomes of mechanically ventilated intensive care unit (ICU) patients.

## METHODS

We followed the Preferred Reporting Items for Systematic Reviews and Meta-analyses (PRISMA) statement^27^ and the Cochrane Handbook^28^ for the design, method and presentation of the results of this systematic review and meta-analysis.

### Database search

In this systematic review and meta-analysis, we searched the PubMed, Embase, Cochrane Library and Web of Science databases. The following keywords were used for the search: “oxygen inhalation therapy”, “liberal*”, “conservative*”, “conventional*”, “respiration”, “artificial” and “mechanical ventilation”. We set the publication type to clinical trial only, and the publication language was limited to English. We searched for related literature from the time of database inception up to and including July 25, 2021. The search strategy is presented in Appendix 1.

### Study selection

Two authors independently assessed all titles and abstracts for inclusion and then assessed the full texts of the studies considered.

The studies included had to satisfy the following criteria.

1. The trial needed to have been designed as a clinical control study.
2. The study subjects needed to be adult patients (aged > 18 years) requiring MV.
3. The studies needed to compare liberal and conservative oxygen therapies. We defined conservative oxygen therapy as having a target blood oxygen saturation of 90%-97%.^29,30^ The treatment arm (liberal oxygen therapy) was defined as having a higher oxygen target, measured through any of the following: fraction of inspired oxygen (FiO_2_), arterial partial pressure of oxygen (PaO_2_), arterial oxygen saturation or peripheral oxygen saturation (SpO_2_).
4. The all-cause mortality and number of deaths during the follow-up period needed to be reported in the results. We excluded studies on patients younger than 18 years or patients who were pregnant, along with studies limited to patients with chronic respiratory diseases or psychiatric diseases, patients on extracorporeal life support or patients treated with hyperbaric oxygen therapy or elective surgery. Observational and preclinical studies were also excluded.

### Outcomes

The primary outcome of interest in the current analysis was 28-day mortality. The secondary outcomes analyzed included 90-day mortality, the rate of new infections, ICU length of stay, mechanical ventilation-free time within 28 days and vasopressor-free time within 28 days.

### Data extraction and quality evaluation

Two authors independently screened the studies, extracted data and conducted quality assessments. When agreement could not be reached, the first two authors discussed the decision to include or exclude studies, until an agreement was reached. Two authors extracted and recorded the authors, publication year, study design, participants and population, demographic characteristics, baseline characteristics, details of intervention treatment (oxygen therapy), outcome measurements and results from each enrolled study. The risk of bias in the studies included was evaluated in accordance with the Cochrane risk of bias tool.^31^ The following characteristics were assessed: random sequence generation, allocation concealment, blinding of participants and personnel, blinding of outcome assessments, incomplete outcome data, selective reporting and other bias. For each characteristic, the risk of bias was rated as low, high or unclear (in cases in which there were insufficient details). Two authors independently assessed the study quality, and disagreements were resolved via discussion.

### Statistical analyses

The statistical analysis was accomplished using the Cochrane systematic review software: Review Manager (RevMan) [Computer program], version 5.3 (The Nordic Cochrane Centre: Copenhagen, Denmark). Measurement data were expressed as means and standard deviations and 95% confidence intervals (95% CIs). Enumeration data were expressed as risk ratios (RRs) and 95% CIs. Assessment of heterogeneity was completed using the chi-square test. The I^2^ statistic was used in order to determine the degree of heterogeneity. If the heterogeneity was determined to be low or moderate (I^2^ < 50%; P < 0.1), the fixed-effect model was applied. Otherwise, the random-effects model was used. In the presence of heterogeneity, to eliminate the influence of individual studies, especially small-sample and low-quality studies, leave-one-out sensitivity analysis was conducted.

## RESULTS

### Studies retrieved and included

**Figure 1.**
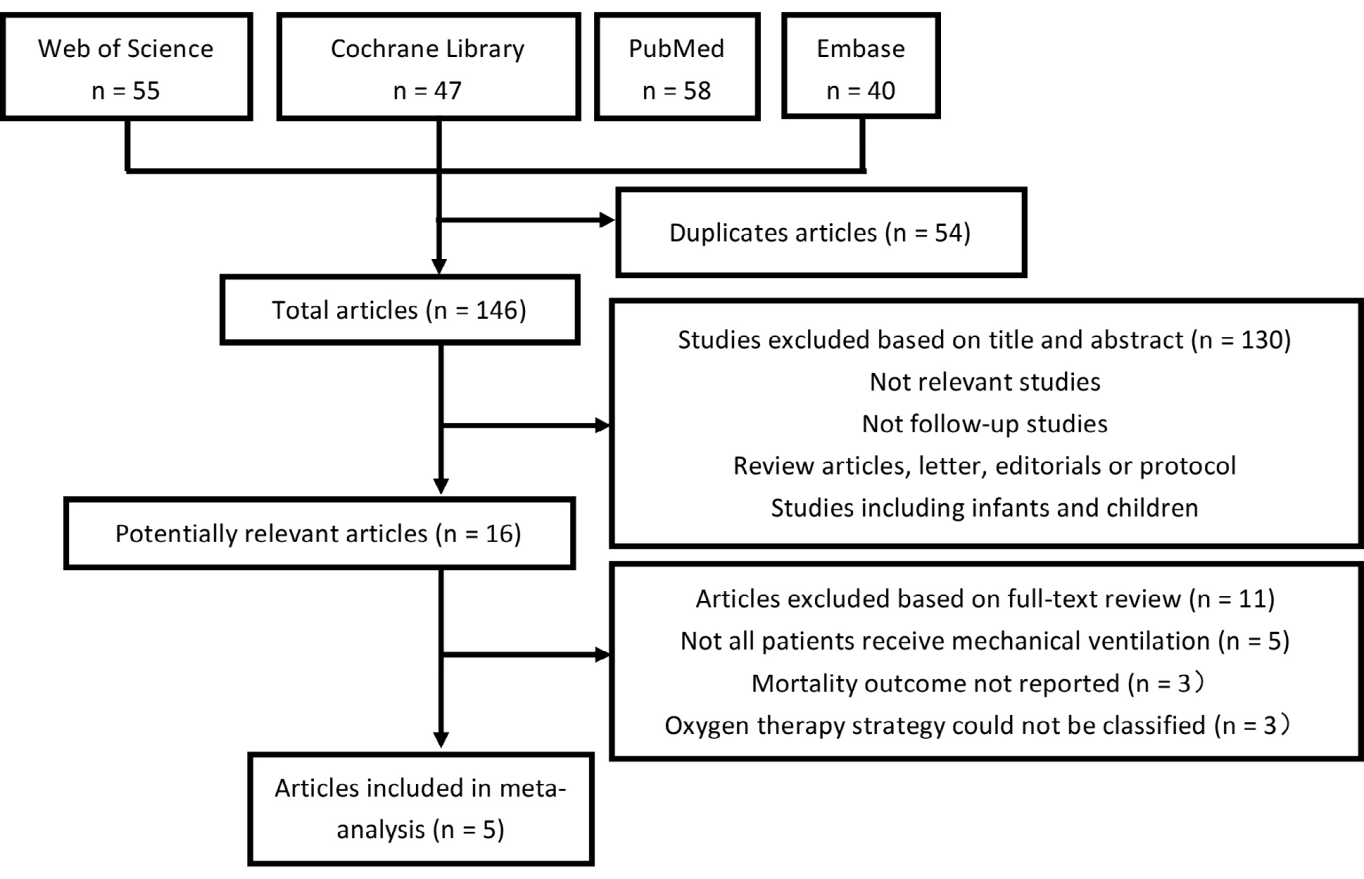
Flow diagram.

We identified 200 studies from PubMed, Embase, Cochrane Library and Web of Science. After screening the titles and abstracts, 16 studies were included for full-text review. In three of the studies, some patients were not mechanically ventilated. In two studies, the number of deaths and mortality rate were not reported. In three other studies, oxygen therapy strategies could not be classified. In the end, four randomized controlled trials (RCTs) and one cohort study^17,23-26^ were included in the meta-analysis (Figure 1).

### Study characteristics and quality evaluation

**Figure 2.**
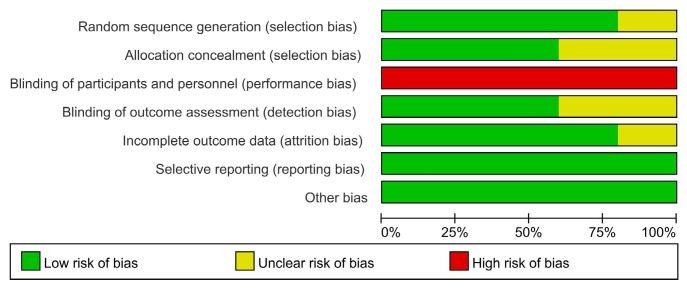
Risk-of-bias graph.

The main characteristics of the eligible RCTs and cohort study are shown in Table 1. Five studies and 1806 mechanically ventilated ICU patients were included in the meta-analysis. The quality of the studies included in this meta-analysis was medium. The quality of the studies included, as assessed using the Cochrane risk-of-bias tool is shown in Figure 2. Because the interventions needed the cooperation of doctors, there was a lack of use of blinding methods. As such, there may have been some bias during implementation of the interventions.

**Table 1.** Characteristics of the studies included

Study	Design	Characteristics												
		Conservative group						Liberal group						
		Sample size, n	Mean age, years		Men, n (%)	PaO_2_/FiO_2_, mean (SD)	APACHE III score mean (SD)	Sample size, n	Mean age, years		Men, n (%)		PaO_2_/FiO_2_, mean (SD)	APACHE III score mean (SD)
Asfar et al.^26^	Randomized controlled trial	217	66·3		140 (65%)	228 (103)	N/A	217	67·8		137 (63%)		220 (103)	N/A
Barrot et al.^25^	Randomized controlled trial	99	63.0		65 (65.7%)	116.8 (47.4)	66.9 (13.7)	102	63.5		64 (62.7%)		120.1 (53.6)	67.9 (14.4)
Mackle et al.^24^	Randomized controlled trial	479	58.1		306 (63.2%)	259 (146)	N/A	480	57.5		302 (62.8%)		245 (138)	N/A
Panwar et al.^23^	Randomized controlled trial	52	62.4		32 (62%)	248 (112)	77.5 (23.6)*	51	62.4		33 (65%)		247 (113)	67.9 (25.6)*
Suzuki et al.^17^	Cohort study	54	54		32 (59%)	302 (142)	68 (32)*	51	51		38 (75%)		278 (137)	68 (39)*

Study	Participants			Interventions						Changes in SpO_2_, PaO_2_ after Interventions				
										Conservative group			Liberal group	
										SpO_2_ mean (SD), %		PaO_2_ mean (SD), mmHG	SpO_2_ mean (SD), %	PaO_2_ mean (SD), mmHG
Asfar et al.^26^	Septic shock patients receiving mechanical ventilation in the ICU			Normoxia group: Target oxygen saturation 88%-95% Hyperoxia group: Mechanical ventilation with FiO_2_ of 1.0 for 24 h after inclusion. Thereafter target as in the normoxia group						N/A		N/A	N/A	N/A
Barrot et al.^25^	ARDS patients receiving mechanical ventilation in the ICU			Conservative-oxygen group: Target oxygen saturation 88%-92% Liberal-oxygen group: The SpO_2_ was maintained at a level of at least 96%						93.16 (0.45)*		71.5 (3.05)*	97.22 (0.45)*	101.9 (4.47)*
Mackle et al.^24^	Patients receiving mechanical ventilation in the ICU			Conservative-oxygen group: The SpO_2_ was maintained between 90% and 97% Liberal-oxygen group: no restrictions						N/A		N/A	N/A	N/A
Panwar et al.^23^	Patients requiring invasive mechanical ventilation in the ICU			Conservative-oxygen group: Target SpO_2_ of 88-92% Liberal-oxygen group: Target SpO_2_ of ≥ 96%						93.5 (0.45)		70 (2.5)	96.8 (0.5)	92 (3.5)
Suzuki et al.^17^	Patients receiving mechanical ventilation in the ICU			Conservative oxygen therapy: The SpO_2_ was maintained between 94% and 97% Conventional oxygen therapy: The SpO_2_ was maintained between 97% and 99%						95.4 (0.48)*		82.5 (9.5)*	97.8 (0.31) *	106.8 (8.5)*

ARDS = acute respiratory distress syndrome; ICU = intensive care unit; SpO_2_ = arterial saturation of peripheral oxygen; PaO_2_ = arterial partial pressure of oxygen; SD = standard deviation; N/A = not applicable; *Estimated values

### Outcomes

#### Primary outcomes

**Figure 3.**
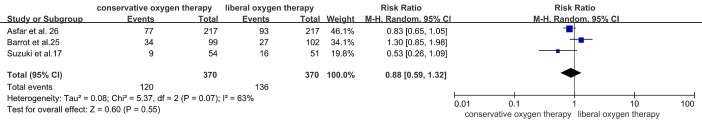
28-day mortality.

**Figure 4.**
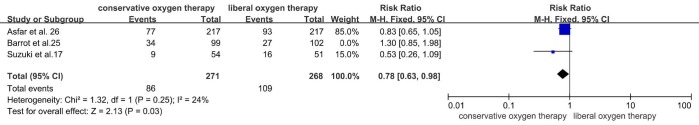
Adjusted 28-day mortality.

Short-term mortality is shown in Figure 3. Three studies^17,25,26^ provided data regarding 28-day mortality. Since there was high heterogeneity among the studies (P = 0.07; I^2^ = 63%), the random-effects model was adopted. The result showed that there was no statistical significance in 28-day mortality between the conservative and liberal groups (RR = 0.88; 95% CI = 0.59-1.32, P = 0.55). Sensitivity analysis was performed to evaluate the effect of a single study on the overall estimate by sequentially excluding each study. The heterogeneity decreased significantly (I^2^ = 24%; P = 0.25). After excluding one of the studies^25^ and making adjustments, oxygen therapy strategy was found to be significantly associated with 28-day mortality, such that the conservative group performed better than the liberal group (RR = 0.78; 95% CI = 0.63-0.98; P = 0.03) (Figure 4).

M-H = Mantel-Haenszel; CI = confidence interval.

M-H = Mantel-Haenszel; CI = confidence interval.

#### Secondary outcomes

**Figure 5.**
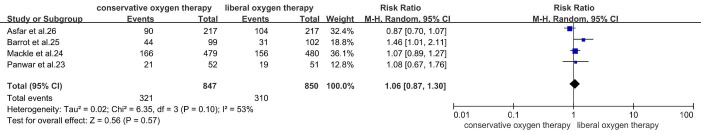
90-day mortality.

Medium-term mortality is shown in Figure 5. Four studies^23-26^ provided data regarding 90-day mortality. As there was high heterogeneity among the studies (P = 0.1; I^2^ = 53%), the random-effects model was adopted. The result showed that there was no statistically significant difference in 90-day mortality between the conservative group and the liberal group (RR = 0.98; 95% CI = 0.85-1.44; P = 0.82).

M-H = Mantel-Haenszel; CI = confidence interval.

**Figure 6.**
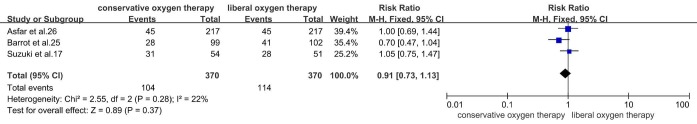
New infection rate.

New infections are shown in Figure 6. Three studies^17,25,26^ provided data regarding the rate of new infections. Since there was no significant heterogeneity among the studies (P = 0.28; I^2^ = 22%), the fixed-effect model was adopted. The result showed that there was no statistically significant difference in the rate of new infections between the conservative and liberal groups (RR = 0.91; 95% CI = 073-1.13; P = 0.73).

M-H = Mantel-Haenszel; CI = confidence interval.

**Figure 7.**
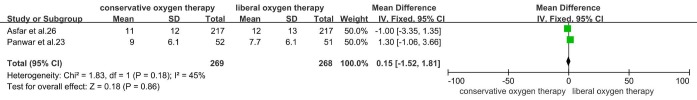
Intensive care unit length of stay.

ICU length of stay is shown in Figure 7. Two studies^23,26^ provided data regarding ICU length of stay. As there was no significant heterogeneity among the studies (P = 0.18; I^2^ = 45%), the fixed-effect model was adopted. The result showed that there was no statistically significant difference in the ICU length of stay between the conservative and liberal groups (mean difference, MD = 0.15; 95% CI = -1.52-1.81; P = 0.86).

SD = standard deviation; IV = inverse-variance weighting; CI = confidence interval.

**Figure 8.**
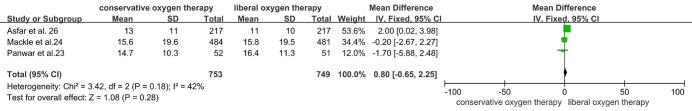
Mechanical ventilation-free time within 28 days.

The mechanical ventilation-free time within 28 days is shown in Figure 8. Three studies^23,24,26^ provided data regarding the mechanical ventilation-free time within 28 days. Since there was no significant heterogeneity among the studies (P = 0.18; I^2^ = 42%), the fixed-effect model was adopted. The result showed that there was no statistically significant difference in mechanical ventilation-free time within 28 days between the conservative and liberal groups (MD = 0.8; 95%; CI = -0.65-2.25; P = 0.28).

SD = standard deviation; IV = inverse-variance weighting; CI = confidence interval.

**Figure 9.**
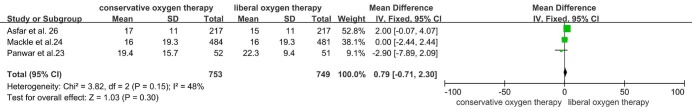
Vasopressor-free time within 28 days.

The vasopressor-free time within 28 days is shown in Figure 9. Three studies^23,24,26^ provided data regarding the vasopressor-free time within 28 days. Since there was no significant heterogeneity among the studies (P = 0.15; I^2^ = 48%), the fixed-effect model was adopted. The result showed that there was no statistically significant difference in vasopressor-free time within 28 days between the conservative and liberal groups (MD = 0.79; 95% CI = -0.71-2.30; P = 0.3).

SD = standard deviation; IV = inverse-variance weighting; CI = confidence interval.

**Figure 10.**
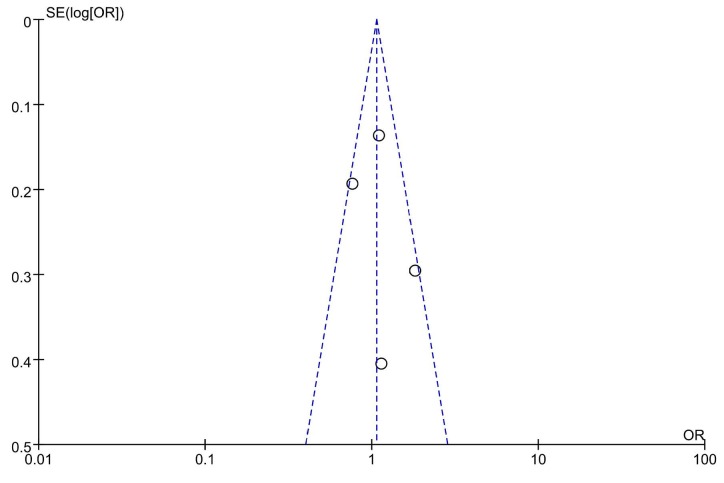
Funnel plot for primary outcome.

The risk of bias in the studies included is shown in Figure 10. The funnel plot of the result showed that the primary outcome was symmetrical. Hence, there was no evidence of significant small-sample effects or publication bias.

SE = standard error; OR = odds ratio.

## DISCUSSION

This systematic review and meta-analysis enrolled 1806 mechanically ventilated ICU patients. All the studies included were considered to be of high quality. Despite the high heterogeneity, the results suggest that conservative oxygen therapy does not increase the risks of short-term mortality, medium-term mortality, new infections, longer ICU length of stay, shorter mechanical ventilation-free time within 28 days or shorter vasopressor-free time within 28 days, for mechanically ventilated ICU patients.

In clinical practice, oxygen therapy has been widely used to prevent or correct arterial hypoxemia for mechanically ventilated ICU patients. Due to concerns over the possible adverse outcomes of hypoxia exposure among critically ill patients, liberal oxygen therapy and hyperoxia are widely used for mechanically ventilated ICU patients. One study reported that 59% of patients have oxygen saturation greater than 98% most of the time.^32^

However, according to the formula of oxygen delivery (DO_2_ = cardiac output × arterial oxygen content; arterial oxygen content = (Hb × 1.34 × SaO_2_) + (0.0031 × PaO_2_)), oxygen delivery is governed by three key factors: arterial saturation (SO_2_), cardiac output (CO) and hemoglobin (Hb). It is unreasonable to only use SaO_2_ as the indicator for evaluating gas exchange in hypoxemic patients. Moreover, the oxygen dissociation curve of hemoglobin is “S-shaped”: the upper part of the curve is very gradual, which means that it is very difficult to further increase SaO_2_ by increasing blood oxygen content and PaO_2_ in the upper part. For example, even when the patient’s PaO_2_ is increased, at the risk of hyperoxia exposure, from 100 mmHg to 150 mmHg, only an incremental increase (200 ml/l to 201.5 ml/l) in the blood oxygen content results from this.^9^ It has also been reported that hyperoxia results in decreased heart rate, reduced CO and increased vascular resistance.^33^

Therefore, liberal oxygen therapy that only focuses on arterial oxygen saturation when increasing the oxygen delivery is unhelpful. Hyperoxia caused by liberal oxygen therapy may even be harmful. It can promote production of reactive oxygen species and expression of inflammatory cytokines, thus increasing the risk and severity of pneumonia,^11^ epithelial and endothelial damage^13^ and pulmonary interstitial edema.^12^

The results from the meta-analysis confirm that in acutely ill patients, liberal oxygen therapy is unhelpful and does not improve patient outcomes, but may increase mortality. When the range of SpO_2_ is more than 94-96%, patients may be affected adversely.^4^ Recent studies have shown that conservative oxygen therapy has no significant adverse effect on ICU patients with respiratory failure and hypoxic ischemic encephalopathy.^34,35^ Conservative oxygen therapy is relatively safe for critically ill ICU patients.

Thus, oxygen therapy should be restricted. The goal of oxygen therapy should be to ensure adequate oxygen delivery while minimizing any unnecessary hyperoxia exposure. However, the question is how conservative it should be. The ideal situation is that supplemental oxygen administration should be guided through assessment of tissue oxygen delivery and consumption. However, these two parameters are difficult to obtain in clinical practice. In clinical trials, conservative oxygen therapy is usually carried out by keeping SpO_2_ at the lower limit of normality.^24,25^ To define conservative oxygen therapy solely on the basis of SpO_2_ seems to ignore assessment of oxygen consumption.

As surrogate parameters for oxygen consumption, blood lactate concentration, central venous-to-arterial CO_2_ difference and central venous or mixed venous oxygen saturation can also help in implementation of conservative oxygen therapy. Over recent years, there has been a conservative trend in oxygen therapy practice in some hospitals.^36^

However, for mechanically ventilated critically ill ICU patients, there is a lack of consensus and explicit guiding criteria regarding the use of conservative oxygen therapy. Clinicians who worry about hypoxemia will still increase the patient’s oxygen saturation as much as possible, even at levels exceeding what they think is reasonable,^8^ even though these clinicians are aware of the potential harm of liberal oxygen therapy.

The results from our study on mechanically ventilated ICU patients showed that there was no significant difference in clinical prognosis between use of liberal and use of conservative oxygen therapies. Conservative oxygen therapy did not result in additional risk; therefore, it is feasible and safe. It is worth mentioning that there was great heterogeneity regarding 28-day mortality among the studies reviewed here. By excluding each study one by one, we found that the heterogeneity arose from the study by Barrot et al.^25^ Excluding Barrot’s study decreased the heterogeneity (I^2^ = 24%).

After adjustments, the results from the meta-analysis revealed that conservative oxygen therapy reduced short-term mortality (RR = 0.78; 95% CI = 0.63-0.98; P = 0.03) (Figure 4). The reasons for this may have been related to the fact that the study population comprised acute respiratory distress syndrome (ARDS) patients. Such patients are characterized by difficult-to-correct hypoxemia. Hypoxemia arises from a diverse range of factors.^37^ There may be little difference in clinical prognosis between liberal and conservative oxygen therapy use until the pathological basis of ARDS has been effectively improved. Moreover, the target for conservative oxygen therapy in the studies reviewed here was set at 88%-92% blood oxygen saturation, which is close to the lower limit recommended in ARDS guidelines.^38,39^ In practice, there was some deviation between the actual and target oxygen saturation. This would undoubtedly have increased the risk of hypoxia exposure in the conservative group. The adverse events of mesenteric ischemia seen in the conservative group may indicate that conservative oxygen therapy close to the lower limit recommended may have been inappropriate.

Furthermore, apart from three studies^17,26^ that only partially included patients with ARDS and the sample population in the study of Barrot et al.,^25^ all the patients included were classified as presenting ARDS. The results suggest that there may have been a discrepancy between ARDS patients and non-ARDS patients regarding the prognosis from conservative oxygen therapy. In other words, this could imply that conservative oxygen therapy is beneficial for reducing short-term mortality among mechanically ventilated patients who do not present ARDS.

### Limitations

The findings reported in this study must be interpreted with caution because of several limitations. Firstly, the definitions of conservative oxygen therapy and liberal oxygen therapy were not quite concordant in the studies that we enrolled, and this may have led to inaccuracies in the relative mortality rates between the conservative and liberal groups. Secondly, the number of studies included was relatively small and, therefore, subgroup analysis according to ARDS status was not possible. Thirdly, we assumed that the respiratory function of mechanically ventilated patients in ICUs would be severely impaired. However, some patients received MV for extrapulmonary reasons, and it was not possible to exclude these patients.

## CONCLUSIONS

Liberal oxygen therapy and higher SpO_2_ for mechanically ventilated ICU patients are not necessary. For partial MV patients, conservative oxygen therapy was not associated with a statistically significant reduction in mortality.

## Appendix 1 Search strategy

Search strategy for PubMed ((“Oxygen Inhalation Therapy”[Mesh]) AND (((liberal*) OR (conservative*)) OR (conventional*))) AND (((Respiration, Artificial[MeSH Terms])) OR (mechanical ventilation)) AND (clinicaltrial[Filter]) Search strategy for Embase (‘oxygen therapy’/exp OR ‘oxygen therapy’) AND (liberal* OR conservative* OR conventional*) AND ((‘respiration,’/exp OR respiration,) AND artificial OR (mechanical AND (‘ventilation’/exp OR ventilation))) AND ‘controlled clinical trial’/de Search strategy for Cochrane library ((liberal*):ab OR (conservative*):ti,ab,kw OR (conventional*):ti,ab,kw) AND ((Respiration, Artificial):ab OR (mechanical ventilation):ti,ab,kw) AND (Oxygen Inhalation Therapy) Search strategy for Web of Science (TS= (Oxygen Inhalation Therapy) AND ((AB=(liberal*) OR AB=(conservative*)) OR AB=(conventional*))) AND ((TS=(Respiration, Artificial)) OR TS= (mechanical ventilation))
